# Anti-fibrotic Potential of AT_2_ Receptor Agonists

**DOI:** 10.3389/fphar.2017.00564

**Published:** 2017-08-31

**Authors:** Yan Wang, Mark Del Borgo, Huey W. Lee, Dhaniel Baraldi, Baydaa Hirmiz, Tracey A. Gaspari, Kate M. Denton, Marie-Isabel Aguilar, Chrishan S. Samuel, Robert E. Widdop

**Affiliations:** ^1^Department of Pharmacology, Cardiovascular Disease Program, Biomedicine Discovery Institute, Monash University, Clayton VIC, Australia; ^2^Department of Biochemistry and Molecular Biology, Cardiovascular Disease Program, Biomedicine Discovery Institute, Monash University, Clayton VIC, Australia; ^3^Department of Physiology, Cardiovascular Disease Program, Biomedicine Discovery Institute, Monash University, Clayton VIC, Australia

**Keywords:** AT_2_ receptor, compound 21, cardiac fibrosis, renal fibrosis, inflammation

## Abstract

There are a number of therapeutic targets to treat organ fibrosis that are under investigation in preclinical models. There is increasing evidence that stimulation of the angiotensin II type 2 receptor (AT_2_R) is a novel anti-fibrotic strategy and we have reviewed the published *in vivo* preclinical data relating to the effects of compound 21 (C21), which is the only nonpeptide AT_2_R agonist that is currently available for use in chronic preclinical studies. In particular, the differential influence of AT_2_R on extracellular matrix status in various preclinical fibrotic models is discussed. Collectively, these studies demonstrate that pharmacological AT_2_R stimulation using C21 decreases organ fibrosis, which has been most studied in the setting of cardiovascular and renal disease. In addition, AT_2_R-mediated anti-inflammatory effects may contribute to the beneficial AT_2_R-mediated anti-fibrotic effects seen in preclinical models.

## Introduction

The renin angiotensin system (RAS) is one of the most important systems to regulate hemodynamics, blood pressure, and tissue remodeling processes. The RAS is a circulating as well as a local hormonal system (Campbell, 1987) such that local generation of angiotensin occurs in many tissues including the brain, heart, kidney, and vasculature (Te Riet et al., 2015). Various components of circulating and tissue RAS that are important for regulating vascular and cardiac contractility, fluid and electrolyte homeostasis, as well as extracellular matrix (ECM) production have been reviewed elsewhere (Campbell, 1987; Te Riet et al., 2015).

There are two major subtypes of angiotensin receptors, the angiotensin II subtype 1 receptor (AT_1_R) and angiotensin II subtype 2 receptor (AT_2_R) (Herichova and Szantoova, 2013; Karnik et al., 2015). It is well established that activation of AT_1_R by angiotensin II (Ang II) mediates pathophysiological effects such as vasoconstriction, proliferation, fibrosis, oxidative stress, and inflammation (Sadoshima and Izumo, 1993; Ferrario and Strawn, 2006; Whaley-Connell et al., 2013), which occurs in multiple organs including heart, kidney, liver, lungs, vascular smooth muscle, and brain (Mehta and Griendling, 2007; Karnik et al., 2015). On the other hand, activation of AT_2_R is thought to counter-regulate the pathophysiological effects induced by AT_1_R and exert vasodilator, anti-fibrotic, anti-proliferative, and anti-inflammatory effects (Widdop et al., 2003; Jones et al., 2008) as well as natriuretic and antihypertensive effects in renal disease (Carey, 2017). The AT_2_R is also very topical in the context of neuropathic pain as it has recently been reported that an old AT_2_R antagonist has been repurposed to treat neuropathic pain. For general interest, an historical account of this recent discovery is also noted (see Keppel Hesselink and Schatman, 2017). However, the current review will focus on recent evidence for an anti-fibrotic effect due to the pharmacological stimulation of AT_2_R in the context of cardiovascular disease.

## Cardiovascular Disease (CVD)

Cardiovascular disease is the leading cause of morbidity and mortality globally (Moran et al., 2014). While progress is being made in addressing CVD risk factors such as high blood pressure, diabetes, obesity, and high cholesterol (Moran et al., 2014), less therapeutic intervention has been directed at some of the underlying pathological changes occurring in relevant organs. In particular, the ECM is now considered an important site for therapeutic intervention (Rockey et al., 2015).

In the heart, for example, hypertensive heart disease is characterized by myocardial ECM expansion due to excess collagen accumulation (Weber, 1989). Injurious stimuli such as myocardial inflammation, cardiac overload, or cardiomyocyte death may activate pro-fibrotic pathways. Several cell types are involved in this process directly by producing matrix proteins (fibroblasts) or indirectly by secreting fibrogenic mediators (macrophages, mast cells, lymphocytes, cardiomyocytes) that in turn promote fibroblast-mediated ECM production. Transforming growth factor (TGF)-β1 is considered the main pro-fibrogenic mediator and promotes the transdifferentiation of fibroblasts into myofibroblasts that contribute to myofibroblast-mediated collagen synthesis leading to excess collagen complex deposition in the ECM (Tomasek et al., 2002; Rockey et al., 2015). This is one of the key cellular events that drive cardiac fibrosis. The accumulation of collagen (scar tissue) replaces cardiomyocytes that leads to the loss of structural integrity of the myocardium (Weber, 1989; Weber et al., 2013). The distinction between reactive interstititial fibrosis and reparative fibrosis, as occurs following myocardial infraction (MI), is not always well defined (Schelbert et al., 2014). In any case, the consequences of ECM expansion such as increased myocardial collagen deposition in patients results in heart dysfunction (Anderson et al., 1993; Brilla et al., 2000; Diez et al., 2002; Weber et al., 2013; Schelbert et al., 2014). Indeed, it has been estimated that fibrotic diseases contributed to about 45% of mortality in Western countries and may be higher in developing countries (Rosenbloom et al., 2013).

## Targeting RAS as Therapeutic Treatment for CVDs

Angiotensin II subtype 1 receptor blockers (ARBs) and angiotensin converting enzyme (ACE) inhibitors (ACEi) are effective treatments for hypertension based on the concept of blocking the AngII–AT_1_R-axis mediated pathological effects (Karnik et al., 2015). Both treatments are effective in hypertensive patients and their antihypertensive effects appear equivalent (Vijan, 2009; Bavishi et al., 2016), although both ARBs and ACEi exhibit only limited capacity to improve cardiovascular outcome in hypertensive patients beyond blood pressure reductions (van Vark et al., 2012; Bavishi et al., 2016). By contrast, anti-fibrotic effects of ACEi and ARBs were clearly demonstrated in tissue biopsies in small well-controlled trials (Brilla et al., 2000; Diez et al., 2002; Querejeta et al., 2004), which were designed to measure cardiac ECM status (although this is clearly not possible in large outcome trials). Therefore, it was not surprising that many studies subsequently combined ACEi and ARBs (dual RAS inhibition) in the hope that this strategy would maximize any potential cardiovascular remodeling (such as fibrosis reduction) to improve clinical outcomes. However, the impact was in fact the opposite: there was an increased risk of adverse renal events such as hyperkalemia and acute renal failure together with symptomatic hypotension (Yusuf et al., 2008; Messerli et al., 2010; Makani et al., 2013). Indeed, dual RAS inhibition is now contraindicated in most cardiovascular guidelines. Clearly, novel treatments are needed that can exert anti-fibrotic effects alone or in combination with individual RAS inhibitors.

## At_2_R Knock Out and Over-Expression Studies

Initially, there were conflicting reports on the anti-fibrotic effects of AT_2_R deletion on cardiac remodeling evoked by pressure overload, Ang II infusion, or myocardial infarction (MI) that were most likely due to the background mouse strains [see Widdop et al. (2003) for review]. Generally, there is strong evidence demonstrating the protective role of AT_2_R activation since AT_2_R knock out mice exhibited enhanced cardiac perivascular (Akishita et al., 2000), renal (Ma et al., 1998; Chow et al., 2014), and liver (Nabeshima et al., 2006) fibrosis following pro-fibrotic stimuli. Furthermore, cardiac overexpression of AT_2_R was protective against Ang II-induced fibrosis (Kurisu et al., 2003), cardiac hypertrophy in spontaneously hypertensive rats (Metcalfe et al., 2004), and during post-infarct remodeling (Yang et al., 2002; Bove et al., 2004; Isbell et al., 2007; Qi et al., 2012). While detrimental effects of cardiac AT_2_R overexpression have been reported (Nakayama et al., 2005), recent evidence suggests that there is an optimal AT_2_R transgene copy number required to protect against MI-induced cardiac hypertrophy and fibrosis (Xu et al., 2014).

## Direct Pharmacological At_2_R Stimulation is Anti-Fibrotic

The development of the selective nonpeptide AT_2_R agonist compound 21 (C21) (Wan et al., 2004) provided another approach for the understanding of AT_2_R function. Compound 21 is highly AT_2_R-selective (Wan et al., 2004; Bosnyak et al., 2011) although some off target effects such as interference with cellular calcium transport have been reported (Verdonk et al., 2012), albeit at concentrations orders of magnitude greater than its AT_2_R binding affinity, as we have previously discussed (McCarthy et al., 2013). Therefore, C21 can generally be considered as a selective AT_2_R agonist and has been used in this context by many research groups. In a seminal study, Kaschina et al. (2008) reported that following 7 days of treatment with C21, the scarring associated with post-MI remodeling was reduced, which correlated with significantly improved cardiac function (Kaschina et al., 2008). In addition, Gelosa et al. (2009) reported that chronic treatment with C21 reduced kidney inflammation and fibrosis in stroke-prone spontaneously hypertensive rats (SHRSP), although the main focus of this study was on stroke protection (Gelosa et al., 2009). Subsequently, there have been a handful of studies published that clearly show that AT_2_R stimulation using C21 exerts anti-fibrotic effects in hearts of SHRSP (Rehman et al., 2012), following chronic MI in rats (Lauer et al., 2014), in vasculature of rats treated with the nitric oxide synthase inhibitor L-NAME (Paulis et al., 2012), and in lungs during pulmonary hypertension (Bruce et al., 2015). While C21 is generally considered to be AT_2_R selective (Bosnyak et al., 2011), not all these studies used the AT_2_R antagonist to confirm an AT_2_R effect. In addition, renal anti-fibrotic effects of C21 have also been reported in kidneys insulted by doxorubicin (Hrenak et al., 2013) or different forms of diabetic nephropathy (Castoldi et al., 2014; Koulis et al., 2015). Details of all aforementioned studies are provided in **Table 1**. Collectively, these studies document a protective role of the C21–AT_2_R axis against organ fibrosis. Intriguingly, it was recently reported that the AT_2_R may form heterodimers with other class A G-protein-coupled receptors, such as relaxin family peptide receptor (RXFP1) to regulate fibrosis progression, as the anti-fibrotic effects of relaxin in the kidney were actually prevented by genetic or pharmacological inhibition of AT_2_Rs (Chow et al., 2014).

**Table 1:** Summary of anti-fibrotic and related protective effects evoked by chronic treatment with C21.

CVD model	Effect of AT_2_R stimulation by C21	Reference
**Cardiac/vasculature effects**		
Myocardial infarction (MI) in Wistar rats: MI @ 7 days exhibited reduced cardiac function, scar formation, and peri-infarct apoptosis and inflammation	C21 (0.03, 0.3 mg/kg/d IP) for 7 days post-MI: Improved MI-impaired cardiac function (echocardiography and cardiac catheterization); decreased scar (by MRI); Decreased inflammation (mRNA cytokines); and apoptosis (caspase 3, Fas ligand) in peri-infarct zone; C21 effects blocked by PD123319	Kaschina et al., 2008
Stroke-prone SHR (SP-SHR); 13 weeks old @ study end: Exhibited modest fibrosis and inflammation in heart and coronary and aortic vessels	C21 (1 mg/kg/d in chow) for 6 weeks: Prevented vascular fibrosis (coronary and aorta) and stiffness (mesenteric); reduced vascular inflammation and oxidative stress (aorta); Decreased cardiac interstitial and perivascular myocardial collagen; unchanged cardiac MMP2/9; Reduced renal inflammatory/T cell infiltration	Rehman et al., 2012
L-NAME-treated Wistar rat; 16 weeks old @ study end: Exhibited increased aortic wall thickness, stiffness, and fibrosis	C21 (0.3 mg/kg/d PO) for 6 weeks with L-NAME: Partially prevented vascular wall stiffening and fibrosis and reduced pulse wave velocity	Paulis et al., 2012
MI in Wistar rats: MI @ 6 weeks exhibited LV remodeling with increased collagen, TGF-β1, MMM2/9, and decreased TIMP1; associated with impaired function (by echo)	C21 (0.03 mg/kg/d IP) for 6 weeks post-MI: Improved MI-impaired cardiac function (echocardiography); Reduced cardiac interstitial fibrosis and TGF-β1 in LV; Decreased MMP2/9; increased TIMP1 and MMP9/TIMP1 ratio	Lauer et al., 2014
Pulmonary hypertension in Sprague Dawley rats; studied 4 weeks after monocrotaline (MCT): Exhibited increased RV pressure; lung fibrosis; RV fibrosis; and increased lung mRNA for TGF-β1, TNF-α, and IL-1β	C21 (0.03 mg/kg/d IP) for 2 weeks; started 2 weeks after MCT: Improved MCT-impaired RV function; Reversed lung and RV fibrosis; Reversed pro-fibrotic and pro-inflammatory cytokines in lungs (mRNA); C21 effects blocked by PD123319 or MasR antagonist	Bruce et al., 2015

**Disease model**	**Effect of AT_2_R activation**	**Reference**

**Renal effects**		
SP-SHR (4 weeks old) fed high salt diet for ∼8 weeks: Exhibited early development of proteinuria, glomerulosclerosis, and renal fibrosis; later accompanied by brain lesions (by MRI)	C21 (0.75, 5, and 10 mg/kg/d PO) for duration of high salt: Highest C21 dose was effective and delayed brain lesions and delayed proteinuria; Reduced glomerulosclerosis, renal fibrosis, and macrophage infiltration; decreased epithelium/mesenchymal differentiation	Gelosa et al., 2009
Doxorubicin-induced renal toxicity in Wistar rats; studied 4 weeks later: Exhibited decreased glomerular density, increased renal oxidative stress	C21 (0.3 mg/kg/d PO) for 4 weeks post-doxorubicin: Renal fibrosis unchanged; Reduced oxidative stress and restored glomerular density	Hrenak et al., 2013
Zucker diabetic fatty rats; 20 weeks old @ study end: Exhibited diabetic nephropathy including glomerulosclerosis, albuminuria, and renal fibrosis	C21 (0.3 mg/kg/d IP) for 15 weeks; Reduced renal glomerular, tubulointerstitial, and perivascular fibrosis; Reduced macrophage infiltration, but modest reduction in albuminuria (only for first 6 weeks of C21)	Castoldi et al., 2014
Streptozotocin in ApoE^-/-^ mice (5 weeks old); studied 20 weeks later: Exhibited diabetic nephropathy including glomerulosclerosis, albuminuria, increased pro-fibrotic and pro-inflammatory cytokines	C21 (1 mg/kg/d PO) for 20 weeks post-STZ; Reduced glomerulosclerosis, mesangial expansion, albuminuria; inhibited many markers of oxidative stress, inflammation, and fibrosis; increased MMP2/9	Koulis et al., 2015

## Potential Anti-Fibrotic Mechanisms of At_2_R

A number of anti-fibrotic mechanisms are likely to be associated with the changes evoked by C21 (**Table 1**). The activation of the pro-inflammatory nuclear factor-κBα (NFκB) pathway is a central transcriptional effector of inflammatory signaling. Nuclear factor-κB activation triggers gene transcription of many inflammatory cytokines, chemokines, and vascular adhesion molecules such as TNF-α, IL-1β, and IL-6 in fibrotic hearts (Torre-Amione et al., 1996; Francis et al., 1998; Plenz et al., 1998).

Rompe et al. (2010) were the first to show that C21 could exert a direct anti-inflammatory effect as C21 inhibited NFκB activation leading to reduced TNF-α-mediated IL-6 release from human dermal fibroblasts. The anti-fibrotic effect caused by C21 was consistently associated with reduced inflammatory responses and inflammatory cell infiltration in a variety of animal models/organs (**Table 1**) and in other studies not directly assessing fibrosis (Matavelli et al., 2011, 2015; Sampson et al., 2016). In particular, C21-mediated renal anti-inflammatory effects occurred within 4 days in hypertensive rats (Matavelli et al., 2011) and modestly protected against diabetic nephropathy in a short-term (4 week) model in rats (Matavelli et al., 2015) whereas C21 consistently evoked renal anti-inflammatory and anti-fibrotic effects in a longer term model of diabetic nephropathy in rats (Castoldi et al., 2014) and mice (Koulis et al., 2015). Taken together, these studies suggest that C21 inhibits inflammatory responses during the development of fibrosis via activation of AT_2_R.

TGF-β1 is a major pro-fibrotic factor that plays a key role in the development of tissue fibrosis (Lijnen et al., 2000). TGF-β stimulates fibroblasts to differentiate into pro-secretory myofibroblasts that in turn enhance ECM protein synthesis (Desmouliere et al., 1993; Tomasek et al., 2002; Berk et al., 2007). At the same time, matrix metalloproteinases (MMPs) degrade ECM proteins and this process is tightly controlled by tissue inhibitors of metalloproteinases (TIMPs) (Weber et al., 2013). However, in an injured organ, TGF-β1 upregulates the expression of protease inhibitors such as plasminogen activator inhibitor (PAI)-1 and TIMPs which contribute to ECM preservation (Schiller et al., 2004).

Given that macrophages are a source of TGF-β1, the inhibition of macrophage infiltration via AT_2_R activation could contribute to reduced TGF-β1 stimulation of fibrotic pathways. In addition, direct stimulation of AT_2_R is well known to increase nitric oxide and cyclic guanosine monophosphate (cGMP) levels, particularly in the kidneys (Siragy and Carey, 1996, 1997) and vasculature (see Widdop et al., 2003), noting that decreased cGMP levels following AT_2_R stimulation have also been reported (Karnik et al., 2015). Importantly, *in vivo* treatment with C21 increased NO/cGMP levels in kidneys (Matavelli et al., 2011, 2015) in keeping with a predominant AT_2_R-cGMP stimulatory effect. Interestingly, cGMP was reported to inhibit TGF-β signaling (Gong et al., 2011), thereby providing another mechanism for AT_2_R stimulation to modify fibrosis production. Indeed, a number of the anti-fibrotic effects of C21 already described were associated with marked reductions in TGF-β1 in heart (Lauer et al., 2014), lung (Bruce et al., 2015), and kidney (Matavelli et al., 2011; Koulis et al., 2015), suggesting that the inhibition of the TGF-β1 cascade is a common mechanism of the anti-fibrotic effect caused by AT_2_R activation. As TGF-β1 acutely increased AT_2_R expression in skeletal muscle (Painemal et al., 2013), it is possible that a similar compensatory response to cardiovascular injury contributes to increased AT_2_R expression in CVD, although the role of such interactions on AT_2_R expression during chronic AT_2_R stimulation is not known.

**FIGURE 1:**
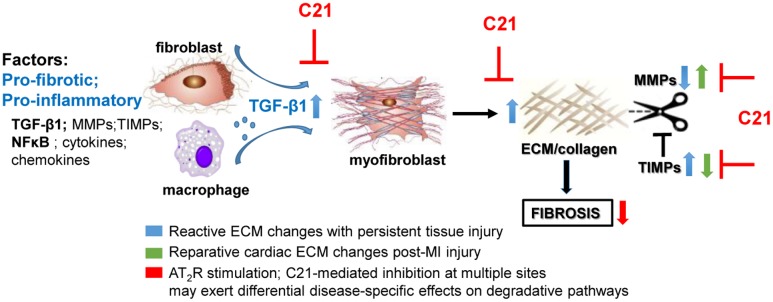
Potential mechanisms involved in the anti-fibrotic actions of AT_2_R stimulation based on the effects of C21 (inhibitory sites in red). AT_2_R stimulation consistently reduces inflammatory and pro-fibrotic factors such as TGF-β1 thereby inhibiting myofibroblast differentiation and ECM production. However, the effects of AT_2_R stimulation on ECM turnover may differ depending on the type of fibrosis/disease model studied. C21 inhibited the proteolytic left ventricular expansion associated with MI-induced injury (green arrows) whereas AT_2_R stimulation is more likely to inhibit ECM preservation (blue arrows) associated with persistent injury (e.g., hypertension), thus facilitating ECM degradation.

In terms of collagen metabolism affecting ECM turnover, the effect of AT_2_R activation on collagen degradation and the regulation of the MMP/TIMP balance is likely to depend on the experimental conditions studied, such as whether the main driver for fibrosis is reparative (in the case of MI) or persistent reactive fibrosis (in the case of hypertensive heart disease). Associated with the anti-fibrotic effect of C21, MMP2/9 levels were either unchanged in SHRSP hearts (Rehman et al., 2012), increased in diabetic murine kidneys (Koulis et al., 2015), or decreased in MI-injured rat hearts (Lauer et al., 2014). These discrepant results are likely to reflect the different requirements of ECM in such models. For example, following MI, cardiac TGF-β1 and MMP levels were elevated whereas cardiac TIMP levels were reduced (Lauer et al., 2014). These somewhat opposing changes caused by MI itself, i.e., pro-fibrotic TGF-β1 activity together with increased proteolytic activity seen by raised MMP-9/TIMP-1 ratio, reflects the need to repair and remodel the heart following MI. In this instance, C21 appears to protect the heart by reducing widespread collagen production (decreased TGF-β1) and attenuating volume expansion (decreased MMP-9/TIMP-1 ratio). By contrast, the ability of C21 to reduce fibrosis in persistent reactive fibrotic models of CVD probably reflects both impaired collagen production (decreased TGF-β1 and collagen), as well as increased degradation due to raised MMP levels (Koulis et al., 2015), which is clearly different to abruptly developing MI-induced cardiac remodeling (**Figure 1**).

## Conclusion and Future Directions

Collectively, these studies demonstrate that pharmacological AT_2_R stimulation evokes decreases in organ fibrosis, most studied in the heart and kidneys to date. The effects of C21 on cardiac ECM remodeling may differ depending on the preclinical fibrotic model studied (**Figure 1**), which is likely to reflect the prevailing circumstances in response to injury, i.e., replacement fibrosis following MI versus persistent reactive interstitial fibrosis seen in hypertensive heart disease. However, AT_2_R stimulation also usually involves an anti-inflammatory effect that may contribute to the beneficial AT_2_R-mediated anti-fibrotic effects. Most data related to chronic AT_2_R stimulation have been obtained using C21, although there are a number of other AT_2_R agonists beginning to emerge in the literature (Jones et al., 2011; Guimond et al., 2014; Del Borgo et al., 2015; Mahmood and Pulakat, 2015) that require rigourous *in vivo* testing in a similar manner to C21. Such studies will shed further light on the clinical potential of AT_2_R agonists in CVD.

## Author Contributions

RW and CS conceived the review; YW wrote the first draft; MDB and DB provided literature searches and contributed to draft. YW, HL, BH, TG, and RW contributed and performed experiments in **Figure 1**. RW, CS, M-IA, and KD did major revisions to the draft manuscript and approved final submission.

## Conflict of Interest Statement

The authors declare that the research was conducted in the absence of any commercial or financial relationships that could be construed as a potential conflict of interest.
